# A Reappraisal of GAT-1 Localization in Neocortex

**DOI:** 10.3389/fncel.2020.00009

**Published:** 2020-02-13

**Authors:** Giorgia Fattorini, Marcello Melone, Fiorenzo Conti

**Affiliations:** ^1^Department of Experimental and Clinical Medicine, Faculty of Medicine and Surgery, Università Politecnica delle Marche, Ancona, Italy; ^2^Center for Neurobiology of Aging, IRCCS INRCA, Ancona, Italy; ^3^Fondazione di Medicina Molecolare, Università Politecnica delle Marche, Ancona, Italy

**Keywords:** GAT-1, GABA transporters, astrocytes, oligodendrocytes, microglia

## Abstract

γ-Aminobutyric acid (GABA) transporter (GAT)-1, the major GABA transporter in the brain, plays a key role in modulating GABA signaling and is involved in the pathophysiology of several neuropsychiatric diseases, including epilepsy. The original description of GAT-1 as a neuronal transporter has guided the interpretation of the findings of all physiological, pharmacological, genetic, or clinical studies. However, evidence published in the past few years, some of which is briefly reviewed herein, does not seem to be consistent with a neurocentric view of GAT-1 function and calls for more detailed analysis of its localization. We therefore performed a thorough systematic assessment of GAT-1 localization in neocortex and subcortical white matter. In line with earlier work, we found that GAT-1 was robustly expressed in axon terminals forming symmetric synapses and in astrocytic processes, whereas its astrocytic expression was more diffuse than expected and, even more surprisingly, immature and mature oligodendrocytes and microglial cells also expressed the transporter. These data indicate that the era of “neuronal” and “glial” GABA transporters has finally come to a close and provide a wider perspective from which to view GABA-mediated physiological phenomena. In addition, given the well-known involvement of astrocytes, oligodendrocytes, and microglial cells in physiological as well as pathological conditions, the demonstration of functional GAT-1 in these cells is expected to provide greater insight into the phenomena occurring in the diseased brain as well as to prompt a reassessment of earlier findings.

## Introduction

γ-Aminobutyric acid (GABA) transporter (GAT)-1 is a highly conserved molecule that is encoded by *SLC6A1* and transports GABA in a high-affinity, Na^+^- and Cl^−^-dependent manner (Kanner, 1978; Guastella et al., 1990; Borden, 1996). As the major GABA transporter in the brain, it plays a key role in modulating GABA signaling (Cherubini and Conti, 2001; Scimemi, 2014). Besides being involved in a broad range of brain functions (Cherubini and Conti, 2001; Bragina et al., 2008; Conti et al., 2011; Kinjo et al., 2013; Scimemi, 2014; Savtchenko et al., 2015; Zafar and Jabeen, 2018), GAT-1 has also been implicated in the pathophysiology of a number of neuropsychiatric disorders including anxiety, depression, epilepsy, Alzheimer’s disease, and schizophrenia (Lai et al., 1998; Nägga et al., 1999; Pierri et al., 1999; Sundman-Eriksson and Allard, 2002; Conti et al., 2004; Lewis and Gonzalez-Burgos, 2006; Cope et al., 2009; Bitanihirwe and Woo, 2014; Carvill et al., 2015; Gong et al., 2015; Fuhrer et al., 2017; Mattison et al., 2018).

GABA uptake by GAT-1 is heavily inhibited by cis-3-aminocyclohexane carboxylic acid (ACHC) and, to a lower extent, by 2,4-diaminobutyric acid, but not by β-alanine (Guastella et al., 1990; Keynan et al., 1992; Liu et al., 1993), two features that have often been considered typical of “neuronal” transporters. This view has been bolstered by the demonstration that GAT-1 is strongly expressed in axon terminals (Minelli et al., 1995; Conti et al., 1998)—despite the fact that the same studies also clearly documented an astrocytic localization—and is still widely used to interpret physiological, pharmacological, genetic, and clinical investigations. However, the findings of several studies published in the past few years call for a more detailed analysis of GAT-1 localization.

## Recent Studies Suggest a Less Simplistic Scenario

After reports of *SLC6A1* variants in patients with myoclonic atonic epilepsy (Dikow et al., 2014; Carvill et al., 2015; Mattison et al., 2018; Cai et al., 2019; Posar and Visconti, 2019), clinical, neurophysiological, and genetic examination of a relatively large cohort of subjects (*n* = 34) bearing *SLC6A1* mutations demonstrated that 97% of them exhibited varying degrees of intellectual disability (ID) and that 91% had been diagnosed with epilepsy (absence, myoclonic, or atonic) based on EEG patterns characterized by irregular, high, ample, generalized spikes, and wave discharges (Johannesen et al., 2018). Notably, more than 60% of these subjects had suffered from moderate or significant ID before epilepsy onset, whereas in a limited number of cases, the ID was not accompanied by epilepsy. Although genetic analysis of the *SLC6A1* variants suggested that the probable disease mechanism was loss of GAT-1 function, assessment of the clinical characteristics associated to them disclosed a wide phenotypic spectrum where the dominant sign, ID, is not quite a “pure” neuronal disorder (Di Marco et al., 2016; Iwase et al., 2017; Maglorius Renkilaraj et al., 2017).

Earlier this year, Inaba et al. (2019) used a model of chronic brain hypoperfusion to assess the protective effects conferred by the anticonvulsant levetiracetam (LEV) on the white matter of mice subjected to bilateral common carotid artery stenosis (BCAS). They found that LEV: (i) did confer protection against learning and memory impairment and white matter injury; (ii) induced PKA/CREB activation; (iii) raised the number of (GFAP-labeled) astrocytes in a time-dependent manner; (iv) reduced Iba-1-positive (+) microglial cells; and (v) increased oligodendrocytes and their precursor cells (Inaba et al., 2019). According to the evidence published to date, synaptic vesicle protein SV2A is the sole receptor for LEV (Lynch et al., 2004). However, an earlier report that LEV increases GAT-1 expression (Ueda et al., 2007), presumably through protein–protein interactions—as recently shown for other vesicular proteins (Marcotulli et al., 2017)—suggests that at least some of the effects described by Inaba et al. (2019) might be mediated through GAT-1.

In 1990, Braestrup and colleagues reported that tiagabine [(3R)-1-[4,4-bis(3-methylthiophen-2-yl)but-3-en-1-yl]piperidine-3-carboxylic acid] nipecotic acid] binds GAT-1 with high affinity (Braestrup et al., 1990). Subsequently, after GAT-1 cloning and functional characterization (Guastella et al., 1990), tiagabine was demonstrated to interact specifically with it (Borden et al., 1994; Borden, 1996) and to be a clinically effective antiepileptic drug (Suzdak and Jansen, 1995; Schousboe and White, 2009; Froestl, 2011). The selectivity of tiagabine for GAT-1 confines its action to those regions of the central nervous system where the transporter plays a large role (neocortex, cerebellum, and hippocampus; Jasmin et al., 2004). Tiagabine has also been found to exert antinociceptive, anxiolytic-like, sedative, and antidepressant-like actions (Jasmin et al., 2004; Sałat et al., 2015). Finally, tiagabine monotherapy appears to improve the performance of epilepsy patients on a number of neuropsychological tests (Dodrill et al., 1998), an effect that seems to relate to the report that heterozygous mice show greater learning and memory compared to wild-type and homozygous *GAT-1*^−/−^ mice (Shi et al., 2012).

In 2015, two articles revived the interest in the effects of tiagabine. In a study of cerebellar GABA signaling using a mouse model of diffuse white matter injury (DWMI), a severe neurological syndrome characterized by hypomyelination and disruption of subcortical white matter development and involving behavioral, cognitive, and motor deficits, Zonouzi et al. (2015) demonstrated that tiagabine enhances the progression of NG2 (oligodendrocyte precursor) cells and promotes oligodendrogenesis and myelination. The same year, Liu and coworkers documented that in a methyl-4-phenyl-1,2,3,6-tetrahydropyridine (MPTP) mouse model of Parkinson’s disease, tiagabine pretreatment attenuates microglial activation, it confers partial protection on the nigrostriatal axis, and it alleviates motor deficits, but its protective function is abolished in GAT-1 knockout mice challenged with MPTP. The authors also found that tiagabine suppresses microglial activation in mice treated by intranigral lipopolysaccharide infusion, an alternative model of Parkinson’s disease (Liu et al., 2015). Although neither study clarified the mechanism(s) underlying tiagabine’s action, it is conceivable that the effects described by Zonouzi et al. (2015) and Liu et al. (2015) depend on a direct action on GAT-1 expression by microglial cells and oligodendrocytes, which may go some way toward explaining the findings of the two groups.

## Evidence for a Widespread Cellular Expression of GAT-1

**Figure 1:**
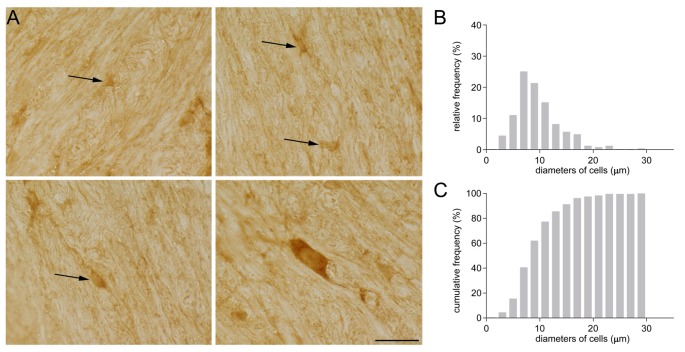
**(A)** GAT-1 immunoreactivity in the subcortical white matter reveals the presence of numerous cells of small and medium size (arrows) and of different morphology. **(B)** Frequency and **(C)** cumulative frequency distribution of the diameter of GAT-1-positive cells. Bar: 20 μm (modified from Fattorini et al., 2017).

Some years ago, while investigating GAT-1 immunoreactivity in subcortical white matter, we detected GAT-1 cells of different sizes and morphologies (Figure 1). Some were small and round with small processes (Figure 1A), and others were medium-sized, rounded or oval with regular profiles; some medium-sized cells had a pyramidal shape with long and intensely stained processes, whereas other cells were large and elongated. The frequency distribution of their diameter is reported in Figure 1B. The broad difference in the size and morphology of these subcortical white matter cells suggested to us that they might belong to different types. We therefore set up a study to examine them in detail.

**Figure 2:**
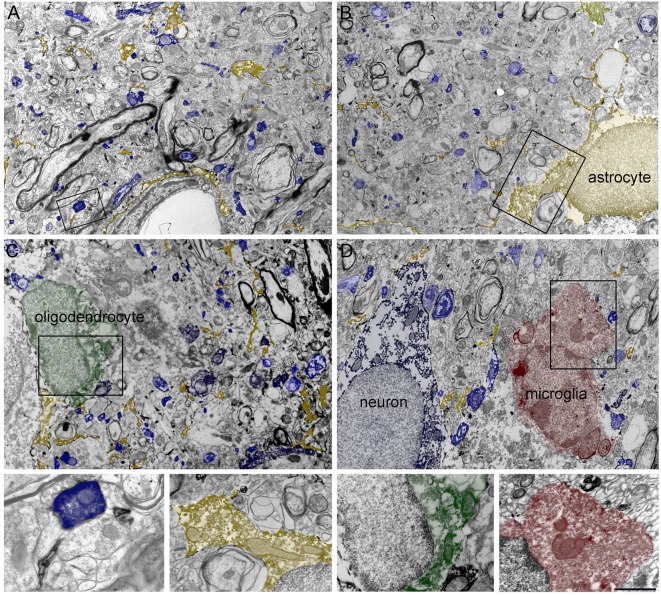
**(A–D)** Four low-magnification electron microscopic (EM) fields showing GAT-1 immunoreactivity in cerebral neocortex (layers II–III of rat parietal cortex). Colored profiles code for different GAT-1-positive cell types and/or profiles: blue, axon terminals, axon, and neuron; yellow, astrocyte and astrocytic processes; green, oligodendrocyte; red, microglial cell. Framed regions in **(A–D)** are reproduced and enlarged, in the lowest portion of the figure. Bar: 2.5 μm for **(A–D)**; 0.8 and 1 μm for enlarged frames of **(A)** and **(B–D)**, respectively (modified from Melone et al., 2015; Fattorini et al., 2017, 2020).

In line with earlier work (Minelli et al., 1995; Conti et al., 1998), electron microscopic (EM) observation demonstrated that GAT-1 was robustly expressed in axon terminals forming symmetric synapses and in astrocytic processes. However, its astrocytic expression was more diffuse than expected and, even more surprisingly, immature and mature oligodendrocytes and microglial cells also expressed the transporter (Figure 2).

### Astrocytes

Recently, quantitative EM analysis, performed in our laboratory, disclosed hitherto unknown features of astrocytic GAT-1 localization in rat cerebral cortex; in particular, we found that: (i) approximately 43% of GAT-1+ profiles in the cortical neuropil are astrocytic processes; (ii) at synaptic loci, GAT-1+ astrocytic processes lie close to the pre- and postsynaptic elements of symmetric as well as asymmetric synapses; and (iii) astrocytic GAT-1 expression at symmetric synapses is not homogeneous, since in ~15% of cases it is associated to GAT-1+ axon terminals and in ~22% of cases it is exclusively localized in astrocytic processes associated to symmetric synapses (i.e., not expressing GAT-1 in axon terminals). The latter fraction of astrocytic GAT-1 increases to up to ~38% in GABAergic synapses targeting distal dendrites and spines, where GAT-1+ axon terminals are less numerous (Melone et al., 2014). Immunogold EM demonstrated that the density of GAT-1 molecules in astrocytic process membranes was ~3.5 times higher than in axon terminals and displayed a continuous distribution from perisynaptic to extrasynaptic regions (respectively within and over 300 nm from the borders of the symmetric synapse specializations), with peaks of concentration at ~950 nm; in contrast, GAT-1 molecules in the membranes of axon terminals showed a preferential perisynaptic localization (Melone et al., 2015).

### Oligodendrocytes

EM analysis revealed GAT-1 immunoreactivity in immature and mature oligodendrocytes both in gray matter and in subcortical white matter. Co-localization studies of GAT-1 and specific oligodendrocyte markers (NG2 and RIP) demonstrated that approximately 12% of GAT-1+ cells in white matter were immature oligodendrocytes and that about 15% were mature oligodendrocytes. Studies of radiolabeled GABA uptake, performed to establish whether GAT-1 localized in oligodendrocytes was functional, demonstrated significant inhibition of Na+-dependent GABA uptake in the presence of tiagabine, indicating that GABA uptake in oligodendrocytes is driven by GAT-1 (Fattorini et al., 2017).

### Microglial Cells

EM analysis also demonstrated GAT-1 immunoreactivity in the soma of microglial cells in subcortical white matter and cortical gray matter as well as in microglial processes, where GAT-1 was localized predominantly in the proximal portion. To quantify GAT-1 protein in microglial cells, we measured the volume of the cells containing the GAT-1 protein signal (in cx3cr1+/gfp animals) and found that it was ~3% in subcortical white matter and ~8% in cortical gray matter. We also established that Na+-dependent GABA uptake was significantly inhibited by NNC-711, a potent GABA uptake inhibitor with high affinity and selectivity for GAT-1 (Borden et al., 1994). In addition, we documented that, like neurons, microglial cells can regulate the membrane expression of GAT-1 in a syntaxin1A-dependent manner (Deken et al., 2000), since syntaxin1A-specific cleavage by botulin toxin C1 (Schiavo et al., 1995; Deken et al., 2000) completely blocks GAT-1-dependent modulation of GABA uptake (Fattorini et al., 2020).

## Discussion

The notion that GAT-1 is not an exclusively “neuronal” transporter appears to be gaining momentum. Indeed, quantitative analysis of GAT-1 in the cerebral cortex, performed in our laboratory, showed that 54% of GAT-1 + profiles were neuronal and that no less than 42% were astrocytic (Melone et al., 2015). More recently, we reported significant GAT-1 expression in oligodendrocytes and microglia (Fattorini et al., 2017, 2020; Figure 2). In this connection, it is worth noting that GAT-3, a putative “glial” transporter (see Minelli et al., 1996 for the neocortex), also seems to be expressed in brainstem and cortical neurons, at least in certain experimental conditions (Clark et al., 1992; Melone et al., 2003, 2005, 2015), and that GAT-2, another putative “glial” transporter, is expressed in epithelial cells and, although at a very low level, also in neurons (Conti et al., 1999). It therefore seems that the era of “neuronal” and “glial” GABA transporters has finally come to a close.

The demonstration that all major brain cells express GAT-1 will conceivably contribute to generate a wider framework through which to assess (and indeed reassess) numerous cerebral GABA-mediated phenomena that occur in physiological conditions. This requires tackling first the issue of the physiological role of GAT-1 in oligodendrocytes and microglial cells. Given the well-established involvement of astrocytes, oligodendrocytes, and microglial cells in pathophysiological conditions (Verkhratsky and Butt, 2013), the demonstration of functional GAT-1 in these cells is expected to provide greater insight into the phenomena occurring in the diseased brain and to prompt a reappraisal of earlier findings. Notably, one of the studies that stimulated the present reassessment (Zonouzi et al., 2015) can now be interpreted as showing that the contribution of GAT-1 to the pathophysiology of DWMI may be mediated by oligodendrocytes, and a similar situation may well arise for the ID seen in some forms of epilepsy. Also, the findings reported by Liu et al. (2015) could simply be interpreted as indicating that GAT-1 expression by microglia may be the direct mechanism by which the transporter contributes to the pathophysiology of Parkinson’s disease.

## Author Contributions

GF, MM, and FC discussed the project, realized the figures, and wrote the article.

## Conflict of Interest

The authors declare that the research was conducted in the absence of any commercial or financial relationships that could be construed as a potential conflict of interest.
